# Morbidity and Mortality in Iranian Children with Juvenile Systemic Lupus erythematosus

**Published:** 2014-07-01

**Authors:** Fariba Tavangar-Rad, Vahid Ziaee, Mohammad-Hassan Moradinejad, Fatemeh Tahghighi

**Affiliations:** 1 Growth and Development Research Center; 2 Pediatric Rheumatology Research Group, Rheumatology Research Center; 3 Department of Pediatrics, Tehran University of Medical Sciences; 4 Children’s Medical Center, Pediatrics Center of Excellence, Tehran, Iran

**Keywords:** Systemic Lupus Erythematosus, Mortality, Morbidity, Juvenile, Children, Iran

## Abstract

***Objective:*** Juvenile systemic lupus erythematosus (JSLE) is a common rheumatologic disorder that involves multi organ systems. Prognosis of lupus in children may be poorer than in adults. In this study, we determined mortality and morbidity rates in the pediatric SLE in Iran.

***Methods:*** In a cross sectional study, we evaluated 120 children with SLE who had registered in our pediatric rheumatology database within 2004-2010. Data including sex, age, remission, age at the time of diagnosis, age at the time of study, various types of organ involvement, mortality and remission were extracted from this database.

***Findings*** ***:*** From 120 cases, 77% (92 cases) were females and 23% (28 cases) males (F:M=3.3). Mean follow up period was 56±32 months and mean age at the time of manifesting disease 10.34±2.9 years. Mortality rate was 10% (12 cases) in our investigation. Musculoskeletal involvement showed significant difference between various age groups (*P*<0.01), that was more frequent in group of 7 years and older. Frequency of oral ulcer and ophthalmic involvement in boys was significantly higher than in girls (*P*<0.05). Frequency of cardiovascular involvement (*P*<0.01) and renal involvement (*P*<0.01) was significantly higher in the patients who had no remission. There was a significant association between mortality rate and cardiac (*P*<0.02, OR=4.9), pulmonary (*P*<0.01, OR=10.167) and liver (*P*<0.05, OR=1.19) involvement.

***Conclusion:*** In our investigation 1-year survival rate was 97% and 5-year survival rate 89%. Liver, cardiac and pulmonary involvements have an association with higher mortality in JSLE patients.

## Introduction

Juvenile Systemic Lupus erythematosus (JSLE) is a common rheumatologic disease with a global incidence rate of 0.28-0.9 per 100,000 population and prevalence rate of 500 cases in 1 million people^[^^1^^,^^2^^]^. Incidence rate of JSLE differs in various racial and ethnical populations and is more prevalent in Asian populations^[^^3^^]^. This disease is able to involve numerous organ systems such as cardiovascular, renal, hematological, musculoskeletal, neurological and mucocutaneous systems^[^^2^^,^^4^^,^^5^^]^. 

 Although SLE mostly occurs in the ages of 20-40 years, however it can occur in all age groups including childhood and its prognosis will be better with increasing age^[^^6^^]^. Prognosis in children in comparison with adults is poorer, probably related to more frequent renal and neurological involvement in JSLE^[^^7^^]^. Several studies have shown that severity and tissue damage in children is higher than in adults with SLE and majority of these complications occur within the first years after manifestation of the disease^[^^5^^-0^^]^. 

 SLE is a chronic disease with a various severity from a mild prolonged to an acute life threatening disease. In the past years pediatric lupus was considered as a fatal disease, but nowadays its clinical course and prognosis has become better in children; so that, 5-year survival is now more than 90%^[^^9^^]^.

 Since clinical manifestations and outcomes are different in various racial and ethnical groups, we designed this study to determine mortality and morbidity rates in Iranian children with systemic lupus erythematosus.

## Subjects and Methods

In this cross sectional study, we extracted data from our pediatric rheumatology registry. All JSLE patients who had been diagnosed between 2004- 2010 were selected for this study. Our center is a tertiary pediatric medical center in Iran and it is one of the two pediatric rheumatology centers in Iran. All patients met diagnostic criteria of lupus according to the American College of Rheumatology revised criteria for the classification of systemic lupus erythematosus^[^^10^^]^. Patients were divided into 3 groups according to age at the time of study: less than 7 years, between 7-14 years and more than 14 years. Moreover, demographic data, first clinical manifestation, follow-up period, morbidity and mortality of the patients and family history of rheumatologic diseases were evaluated. 

 All patients were treated with high dose steroid, hydroxychloroquine and/or immunosuppressives based on organ disorder. New cases and patients who had been followed up less than six months were excluded from the study.

 In this study morbidity was considered as a permanent symptom or complication of the disease or treatment and remission was defined as a state or period during which the clinical and laboratory of the disease are abated. 

 This study was approved by ethical committee of Tehran University of Medical Sciences. Data was analyzed by SPSS software ver17. We used Fisher exact test and Chi-2 test for statistical analysis. *P*-value less than 0.05 was considered as significance level.

## Findings

Totally, 120 patients with lupus referred to our center during this study. Female to male ratio was 3.3:1 (92 females and 28 males). Out of these 120 patients, 4 (3.3%) cases were younger than 7, 30 (25%) were 7-14 years and 86 (71.7%) older than 14 years. Mean age at the time of manifestation of disease was 10.34± 2.9 years. Mean follow-up period was 56±32 (range: 12-120) months. All patients have had constitutional symptoms. Joint, mucocutaneous and hematological manifestations are respectively the most common organ involvement. Frequencies of organ involvement have been summarized in Table 1. 

 Joint and bone involvement were more common in patients aged above 7 years at the time of diagnosis and the time of study (*P*=0.01). Indeed, oral ulcer and ophthalmic involvement in boys were significantly higher than in girls (*P*<0.05).

**Table 1:** Frequency of different types of organ involvement in the 120 children with JSLE

**Morbidity**	**Frequency**	**Morbidity**	**Frequency**
**Joint involvement**	91%	Bone involvement	52%
**Mucocutaneous involvement**	90%	Cardiac involvement	51%
**Hematological involvement**	86%	Pulmonary involvement	41%
**Liver dysfunction**	40%	Neurological involvement	39%
**Endocrine involvement**	70%	Ophthalmic involvement	28%
**Renal involvement **	60%	Vascular involvement	24%

JSLE: Juvenile systemic lupus erythematosus

**Table 2:** Frequency of different types of morbidity in 120 patients with JSLE according to disease remission

**Morbidity**	**Remission**		***P.*** ** value**
	**No**	**Yes**	
**Bone and joint involvement **	26.1%	29.2%	0.7
**Gastrointestinal and increase liver transaminases**	26.3%	10%	0.1
**Cardiovascular involvement**	32.7%	7.7%	0.005
**Renal involvement **	37.3%	10.5%	0.001
**Nervous system involvement **	23.3%	22.5%	0.9
**Ophthalmologic involvement **	24%	25%	0.9
**Hematological involvement **	25.5%	18.2%	0.5
**Endocrine involvement **	23.1%	11.8	0.3

JSLE: Juvenile systemic lupus erythematosus

 Generally, 24% of the patients experienced remission. Frequency of different types of morbidity in our patients according to experience of remission is summarized in Table 2. 

 Frequency of cardiovascular and renal involvement was significantly higher in the patients with active diseases (*P*=0.005 and *P*=0.001, retrospectively).

 Mortality rate was 10% (12 cases). Table 3 shows the effect of different variables on mortality rate. According to this table, there is a significant association between mortality rate and cardiac [*P=*0.02, OR=4.90 (1.24-19.63)], pulmonary [*P*=0.01, OR=10.17 (1.24-19.63)] and increased liver transaminases [*P*=0.04, OR=1.19 (1.24-19.63)] involvement. Survival rate was 97% (SD ±0.02) after 1 year and 89% (SD ±0/04) after 5 years. There was no significant difference between morbidity and mortality rates in the groups with and without positive family history.

## Discussion

Systemic lupus erythematosus is a rheumatologic disease that may involve children and adults^[^^2^^]^. Several studies have evaluated mortality and morbidity rate in the children with SLE worldwide^[^^1^^,^^7^^,^^11^^-^^15^^]^. Some authors have found that prognosis in children is poorer than in adults^[^^6^^,^^7^^,^^11^^]^. Investigations in different areas in the world show some differences in clinical features and also in morbidity and mortality rates^[^^1^^,^^4^^,^^6^^,^^12^^,^^15^^,^^16^^]^. 

 We evaluated morbidity and mortality in Iranian children with JSLE. Mortality rate in our series was 10%. This is approximately similar to Gulay's^[^^12^^]^ and Lumina's results^[^^16^^]^, pointing to a mortality rate of 11.5% and 11.8% respectively. In our study no significant difference was found in male and female paticnts. In addition, our results show no significant difference in mortality rate between various age groups, similar to Vachvanichsanong's study^[^^14^^]^.

**Table 3:** The effect of different variables on mortality rate in our patients

**Variables**		**Frequency**	***P*** **-value**
**Gender**	Male	5 (17.9%)	0.1
	Female	7 (7.6%)	
**Age at the time of study**	<7 years	25%	0.6
	7-14 years	10%	
	>14 years	9.3%	
**Age at initiation of the disease**	<7 years	9.1%	0.5
	> 7 years	10.2%	
**Family history for rheumatologic diseases**	No	11 cases (10%)	1
	Yes	1 case (10%)	
**Organ involvement**	Liver (increased liver transaminases)	100%	0.04
	Renal	83%	0.09
	Neurological	82%	0.15
	Cardiovascular	75%	0.02
	Pulmonary	75%	0.01

JSLE: Juvenile systemic lupus erythematosus

 The most frequent organ involvement in the patients who died due to JSLE complications included liver (increased liver transaminases) in 100%, kidney in 83%, nervous system in 82% and heart and lungs in 75% of patients. We found a significant association between mortality rate and liver, cardiac and pulmonary involvement. Similarly, in a study in Mexico, mortality rate was significantly higher in the JSLE patients with cardiac and pulmonary complications^[^^15^^]^.

 Unlike previous reports, in our study liver dysfunction was more common than hepatomegaly (40% vs 13% of the cases). There are few reports on liver involvement in JSLE. Hepatomegaly and liver dysfunction were reported as common findings in active phase of lupus in about 25% and 50% of patients^[^^17^^]^. It can be a clue for disease activity or due to lupoid hepatitis, infection, drug reaction or toxicities and complication of the disease course^[^^18^^,^^19^^]^. In adult patients with SLE, liver dysfunction has been reported in 33-60% of the patients^[^^19^^,^^20^^]^. 

 In our patients 1-year and 5-year survival rate was 97% and 89%, respectively. With early diagnosis and progress in treatment regimens of pediatric lupus with new immunosuppressive drugs (such as Miclofenolate-Mefotile and Cyclosporine) and decrease in prevalence of infections, survival of patients with JSLE has become better during last 3 decades^[^^21^^]^. Similar to other studies 5-year survival in children with JSLE was between 82% to 92%^[^^22^^-^^4^^]^. 

 Reports on frequency of various morbidities have been different^[^^5^^,^^13^^,^^15^^,^^25^^-^^27^^]^. The most common morbidity in our study was joint involvement (91%) that was similar to Marini's study^[^^13^^]^. In our patients joint, skin and hematological involvement were the most common morbidities alike Hui-Yuen's series, in which cutaneous manifestations, arthritis and hematological abnormalities the most common morbidities at the time of SLE diagnosis^[^^28^^]^. Although these findings are similar to those of other researchers, the next most common morbidity in our study was gastrointestinal involvement which is different from Hui-Yuen's study that introduced renal involvement as the next common morbidity. Joint involvement in SLE may be from minor joint pain to severe arthritis. Severe joint deformity or Jaccoud’s arthropathy is a rare finding in JSLE^[^^29^^]^. Overlap of JSLE and juvenile idiopathic arthritis is a rare syndrome with erosive joint involvement which has been named RHUPUS syndrome. Three cases of our patients had RHUPUS syndrome and we reported them in previous report^[^^30^^]^.

 Based on age at the time of diagnosis, only joint involvement showed significant difference between various age groups, being more frequent in the group of 7-year olds and older.

 Alike Descloux'^[^^31^^]^, prevalence of neurological disorders in our study was 38%. In Muscal's patients headache and mood disorders were the most common neurological disorders^[^^25^^]^. In the present series prevalence of headache was 44%. In our previous report on 55 cases, 43% of patients with SLE had lupus headache^[^^32^^]^. 

 In the present study majority of complications occurred more frequently in male gender, only frequency of oral ulcer and ophthalmic complications show statistically significant difference in males and females. Similar to Niaudet^[^^26^^]^ and Vachvanichsanong^[^^14^^]^ findings, we observed that lupus in male gender is associated with higher mortality, frequent hospitalization and poor outcome.

 In this study, having a family with rheumatologic disease had no effect on morbidity or mortality.

 In our patients, frequency of cardiovascular involvement and renal involvement was significantly lower in remission group. On the other hand, one fifth of the patients with cardiac or renal involvement experienced remission compared to the patients without involvement. 

 The sample size of this study was higher than that reported previously. As a limitation of this study, we did not study the causes of death related to heart, lung and kidney. These manifestations may be more related to infection and/or multiple organ failure due to lupus activity and vasculitis. However, physicians should be alert about these organ involvements as a predictive factor for mortality regardless of cause of involvement in JSLE.

## Conclusion

Mortality rate in Iranian JSLE patients was 10%. Liver, cardiac and pulmonary involvement is associated with higher mortality. Follow up with closer intervals is recommended in the cases with liver, heart and pulmonary involvement.
